# The Stressing State Features of a Bottom Frame Structure Revealed from the Shaking Table Strain Data

**DOI:** 10.3390/ma16051809

**Published:** 2023-02-22

**Authors:** Lingxin Zhang, Rui Li, Zijie Shen, Bai Liu, Jianhui Kong, Guangchun Zhou

**Affiliations:** 1 Key Laboratory of Earthquake Engineering and Engineering Vibration, Institute of Engineering Mechanics, China Earthquake Administration, Harbin 150086, China; lingxin_zh@126.com (L.Z.); jianh_kong@163.com (J.K.); 2 Key Laboratory of Earthquake Disaster Mitigation, Ministry of Emergency Management, Harbin 150086, China; 3 School of Civil Engineering, Harbin Institute of Technology, Harbin 150090, China; liubai_12138@163.com (B.L.); gzhou@hit.edu.cn (G.Z.); 4 Key Lab of Structures Dynamic Behavior and Control of China Ministry of Education, Harbin 150090, China; 5 Key Lab of Smart Prevention and Mitigation of Civil Engineering Disasters of the Ministry of Industry and Information Technology, School of Civil Engineering, Harbin Institute of Technology, Harbin 150090, China

**Keywords:** bottom frame structure, strain, seismic, stressing state, mutation, failure load

## Abstract

As a classic issue, structural seismic bearing capacity could not be accurately predicted since it was based on a structural ultimate state with inherent uncertainty. This result led to rare research efforts to discover structures’ general and definite working laws from their experimental data. This study is to reveal the seismic working law of a bottom frame structure from its shaking table strain data by applying structural stressing state theory: (1) The tested strains are transformed into generalized strain energy density (GSED) values. (2) The method is proposed to express the stressing state mode and the corresponding characteristic parameter. (3) According to the natural law of quantitative and qualitative change, the Mann–Kendall criterion detects the mutation feature in the evolution of characteristic parameters versus seismic intensity. Moreover, it is verified that the stressing state mode also presents the corresponding mutation feature, which reveals the starting point in the seismic failure process of the bottom frame structure. (4) The Mann–Kendall criterion distinguishes the elastic–plastic branch (EPB) feature in the bottom frame structure’s normal working process, which could be taken as the design reference. This study presents a new theoretical basis to determine the bottom frame structure’s seismic working law and update the design code. Meanwhile, this study opens up the application of seismic strain data in structural analysis.

## 1. Introduction

Since the 1980s, with the rapid development of many Chinese cities, many buildings with a frame bottom floor and a masonry top floor, called bottom frame structures, have been built. Bottom frame structures are used as buildings on both sides of the street, with the lower floors being used for commercial purposes and the upper floors for work and housing [1]. Compared to frame structures, bottom-frame structures can save 20–25% of construction costs [2]. In general, the bottom frame structures are adapted to the degree of economic development in China and with Chinese characteristics. There are similarities and differences between the bottom frame structure and the European pilotis system. In the pilotis system, the superstructure is usually a shear wall structure, which is grouted and reinforced so that the shear wall can withstand shear forces.

Research on bottom-frame structures began early worldwide, with Mantel [3] arguing that a flexible bottom-floor construction could improve the structure’s seismic performance. As a result, many researchers have conducted experimental and finite element studies on the seismic performance of bottom frame structures. Gao Xiaowang [4,5] conducted seismic tests on the bottom frame structures model with scales of 1/2 and 1/3. Liang Xingwen [6] carried out a proposed dynamic seismic response test of a 1/2 scale model of the double bottom frame. Zheng Shansuo [7] performed three simulated shaking table tests with a 1/6 scale model. All these tests provided a detailed summary of the load-carrying capacity, seismic performance, and damage mechanism of the bottom frame structure under seismic action. As there are many factors affecting the seismic performance of the bottom frame structure, numerical methods are of great importance for the study of the functional performance of the bottom frame structure under seismic conditions. Li Qi [8] conducted a dynamic time analysis of a two-story frame underframe structure and investigated its elastic–plastic response under different seismic effects using the finite element program CANNY. Chen Jun [1] and Song Linbo [9] carried out pushover analysis and elastoplastic time analysis of the underframe structure system by building a finite element model to obtain the densification capacity and elastoplastic response of the underframe structure under seismic action, respectively.

The abovementioned experimental and numerical methods have obtained the damage patterns, load-carrying capacities, and the laws of continuous collapse processes for various types of bottom frame structures under seismic conditions. The characteristics of these experimental and numerical studies can be summarized as follows.

1. the load-carrying capacity of bottom frame structures obtained from these experimental and numerical studies is often the load-carrying capacity corresponding to their ultimate working condition. Moreover, the reference point for the design and construction of the bottom frame structure is also based on the load-carrying capacity corresponding to the ultimate working condition;
2. All of the above studies consider the inherent property of uncertainty/randomness in the ultimate working state of a structure. Design methods based on the ultimate working state are difficult to accurately estimate a structure’s working capacity for various structural and loading conditions. This result has further led to empirical and statistical approaches to structural analysis and design;
3. Data obtained from experimental and numerical studies, such as experimentally measured strains and the strain energy density of the finite element model, are not fully utilized.

Therefore, it is impossible to accurately estimate the seismic load capacity of the bottom frame structure based on existing theories and methods. The final state of the structure contains huge random variations and empirical errors. Therefore, it is impossible to accurately predict a structure’s load-carrying capacity using the ultimate working state as a foothold. Based on this understanding, existing structural analyses do not attempt to reveal the general laws of operation of various structures. Experimental and numerical studies have formed a fixed paradigm [10]. In such a paradigm, the adverse effects of uncertainty in the load-carrying capacity of structures were reduced, resulting in outstanding engineering achievements. However, uncertainty in load-carrying capacity has become a bottleneck in current structural engineering research, and new theories are needed to reveal the laws embedded in the working of structures. Zhou’s view is that Newton’s and Hooke’s laws reveal the transient laws of structural working in the elastic phase, but any theory or law does not reveal the evolution of structures from the elastic–plastic phase to the damage phase. The general laws of structural work may be contained in the experimental strain and displacement data, but new theories and methods are needed to model them to find the laws.

Zhou [11,12] has developed a structural stressing state theory and proposed a corresponding analysis method to break through the above bottlenecks. The structural stressing state theory treats the failure of a structure as an evolutionary process, which can be characterized by modeling the displacement and strain data during the loading process. The elastic-plastic branching points and the starting point of failure are then defined by defining the abrupt change in the evolution of the structure’s stress state. In recent years, the modeling of the stressing state of dozens of structures of different materials and conditions has revealed defined elastoplastic branch points and failure points, including steel box girder bridges [13], arch supports [14], steel-tube-restrained concrete arches [15], steel frames [11], steel nodes [16] and members [17], steel tube [18] and spiral reinforced concrete [19,20] columns, reinforced masonry shear walls [21], and concrete airport pavement [22].

This study proposes a method for modeling the measured strain data from shaking tables of bottom frame structures based on the structural stressing state theory. The measured strain data from the shaking table of the substructure is modeled as a generalized strain energy density (GSED), and the stressing state modes (matrices or vectors) and characteristic parameters of the substructure are established based on the GSED, which are called stressing state characteristic pairs. A mutation determination criterion is applied to determine the location of the mutation points. The mutation points reveal the failure starting point and the elastic–plastic branch point during the seismic damage of the bottom frame structure, and the load corresponding to the failure starting point is defined as the structural failure load.

## 2. The Shaking Table Test of the Bottom Frame Model

### 2.1. The Bottom Frame Model

**Figure 1:**
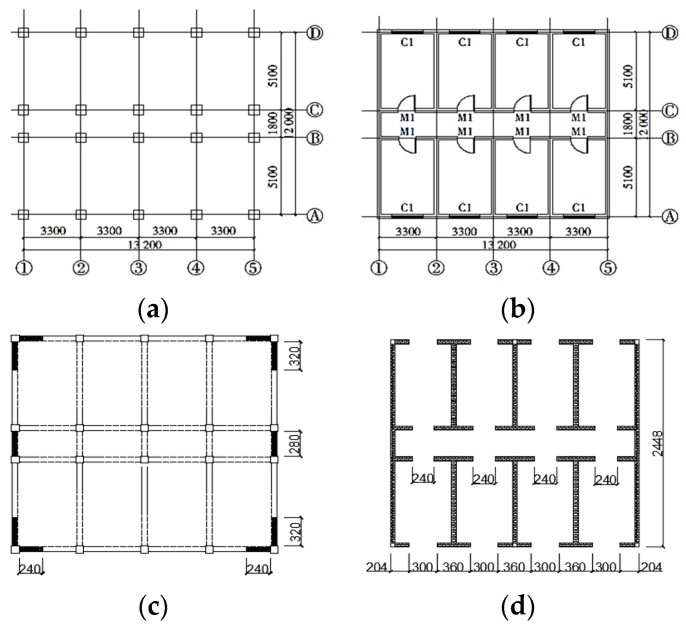
The floor plans of the bottom frame structure: (**a**) the bottom floor plan, (**b**) the standard story plan, (**c**) the anti-seismic wall plan in the bottom story, (**d**) the constructional columns in the upon stories.

**Figure 2:**
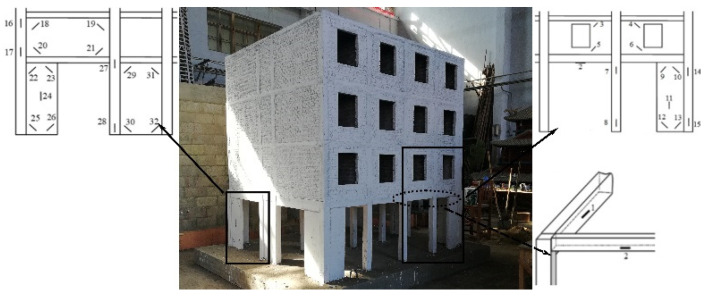
The bottom frame model, the layout of strain gauges and the cracking.

This experimental model in 1/5 scale ratio was designed referring to an actual 4-story bottom frame building close to the street based on the Code for Seismic Design of Buildings of P.R. China GB50011-2010 [23]. The bottom frame was reinforced concrete, and the three stories were masonry structures. The configuration of the structural model is listed in Table 1, and the floor plan is shown in Figure 1. Section 2.3 below shows the accurate picture of the bottom frame model (Figure 2).

The direct application of amplitude-modulated seismic waves to a 1/5 scale model of the bottom frame structure does make it challenging to respond to the non-linear response of the structure and the results from resonance. Therefore, based on the similarity theory, the three seismic waves and the geometrical and material parameters of the model were converted in this study, as shown in Table 2.

The results of the 1/5 scale model shaker tests and the corresponding modeling and study results are equally reliable as long as the similarity theory is sound. In other words, the model tests based on similarity theory concluded that the Eigen-periods of the model were in the ratio of 1/2.23 to that of the actual structure. Therefore, by determining the seismic intensity corresponding to the bottom frame model’s failure point, the actual structure’s seismic eigen-periods can be calculated from the ratio.

The artificial mass of the model is added to simulate the weight and various constant and live loads. For the total mass of real structure: M_T_ = M_beam_ + M_column_ + M_wall_ + M_live_. For the total mass of the model: m_T_ = M_T_ × similarity ratio of mass. For the mass of the model members: m = M_member_ × volume similarity ratio of the model member. For the total mass of artificial weight: m_w_ = m_T_ − m. Table 3 lists the mass parameters of individual stories and the artificial weights.

### 2.2. The Experimental Plan

Table 4 shows the working parameters of the shaking table and the seismic input to test the bottom frame structure model.

Since different seismic inputs considerably affected the experimental output results, the seismic input for the shaking table test selected the typical and prominent seismic records, even the artificial seismic waves consistent with the design response spectrum in the statistical sense. This study selected the El-Centro (El), Taft (Tf), and Wolong (Wl) seismic records according to the practical experience of the shaking table. In order to reflect the possible seismic cases, the test input the three seismic records in the same seismic period (the 30 s), respectively. The horizontal seismic magnitude was applied along the weakest direction. According to Specification 5.1.2 in China Code GB50011-2010, the magnitudes of the Taft wave in three directions were set as X:Y:Z = 1:0.85:0.65. Because the bottom frame model’s mass was not beyond the shaking table’s limit, the similarity ratio of the input seismic accelerations was set as 1. The seismic input scheme is shown in Table 5, in which WNS means the while-noise sweep. Table 5 presents the failure profile, and a description of the bottom frame structure, and the corresponding explanation is given in Section 4.3.

### 2.3. The Experimental Measurement

**Figure 3:**
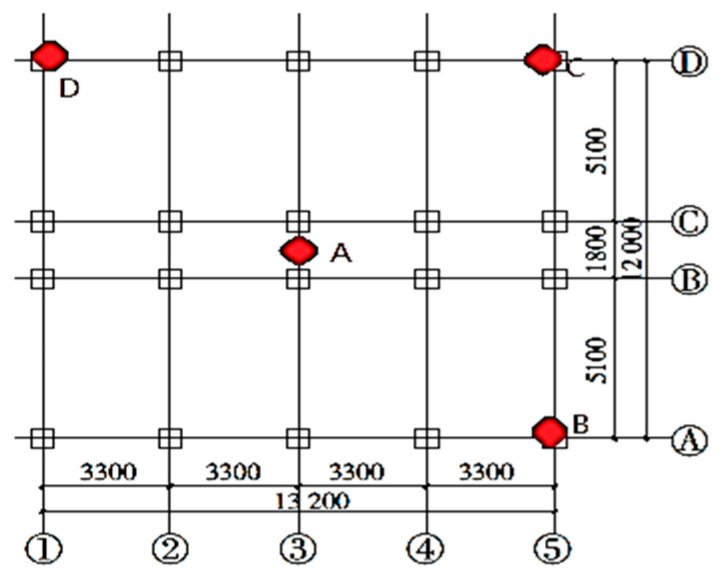
The layout of accelerators and displacement meters.

Figure 2 shows the layout of accelerators and displacement meters on each floor according to China Code GB50011-2010. Twelve accelerators were set to measure the accelerations along with three directions (X, Y, Z). Two horizontal accelerators and one vertical accelerator were put at individual points on the top floor and the ground floor. Two horizontal accelerators were put at the individual points A on the 1st, 2nd, and 3rd floors. Twelve displacement meters were set to measure the displacements along with three directions (X, Y, Z): Two horizontal displacement meters were set at the individual points C on the top and bottom locations of the frame column as well as the second, third, and fourth floors; On the 4th floor, a vertical displacement meter was set at Point B and a lateral displacement meter at Point D to verify the structural torsional response. Figure 3 shows the layout of 32 strain gauges for measuring strains according to China Code GB50011-2010. In addition, three cameras were arranged to picture the cracking propagation on the three sides. It was a pity that only points 2~7 output the stain values in the testing process. However, using the limited strains, structural stressing state theory and method can still present the essential stressing state features of the bottom frame structure.

### 2.4. The Experimental Results

Table 6, Table 7 and Table 8 list the inter-story displacement angles for each level of seismic acceleration used in the shaker tests to describe the evolution of the failure mode of the bottom frame model. It should be noted that first-hand accurate data on the inter-story displacement angles for each level of acceleration were not available for this study, and the inter-story displacement angles for the three types of seismic waves presented in the table are approximations extracted from the data presented in the test report charts for this test.

## 3. Structural Stressing State Theory and Methods

### 3.1. Concept and Physical Law in Structural Stressing State Theory

The structural stressing state manifests structural response at a load level. Structural response data, such as strains and displacements, can be modeled to describe the structural stressing state. The numerical mode (matrix or vector) formed by the response data is called a structural stressing state mode with both shape and magnitude characters so that a parameter can characterize a structural stressing state mode. Stressing state mode and characteristic parameter are called the stressing state characteristic pair.

According to the natural law from quantitative change to qualitative change of a system, the stressing state evolution of a structure with the load increase presents a mutation around a certain load level. This mutation feature is general for various structures under individual loading cases, so it could be called the structural failure law. The law reflects the general and essential working features of structures: (a) Various structures, structural members, and specimens (measuring material strength) undergoing a complete loading process certainly embody the stressing state mutations at specific loads; (b) The stressing state mutations define the failure starting point and the elastic-plastic branch (EPB) point in the structural failure process. Both characteristic points provide physical-law-based references to improving and updating the existing design codes governed by empirical and statistical judgments. In a sense, the structural stressing state theory and the structural failure law could update the foothold (structural ultimate/peak states) of the present structural analysis and design, that is, they could address the classic issue, the uncertainty of structural load-bearing capacity, and structural design.

At present, structural stressing state analysis generally follows the following procedure:

- Model the experimental data to obtain the basic variables to express stressing state modes and characteristic parameters. For instance, this study transforms the tested strains as generalized strain energy density (GSED) values to describe the structural stressing state;
- Build the stressing state characteristic pair of the structure, that is, the stressing state mode and the parameter characterizing the mode.
- Detect the mutation points in the curves of characteristic parameter evolution applying the criterion, and then verify the mutation characteristic in the evolution of the stressing state mode;
- Redefine/update structural failure load and define the EPB load according to structural stressing state mutation features and provide them as the reference to the update and improvement of the existing structural designs.

### 3.2. Modeling of Structural Stressing State

The experimental strains can be modeled as the state variables to express the structural stressing state. However, strains’ directionality makes it challenging to form the stressing state mode (the vector or matrix of strains) and the parameter characterizing the mode. Therefore, a standard method is to model the strain as the scalar quantity:

(1) $$e_{ij}=\int_{0}^{F_{j}}\varepsilon_{i}dF\;\mathrm{or}\;\bar{e_{ij}}=\frac{1}{e_{i,m}}\int_{0}^{F_{j}}\varepsilon_{i}dF$$

where $e_{ij}$ is the GSED values of the ith point at the *j*th load (*F_j_*); $\bar{e_{ij}}$ is the normalized value of $e_{ij}$ ; $e_{i,m}$ is the maximum value among $e_{ij}$ (the load step *j* = 1, 2, …, n); *ε_i_* is the strain at the *i*th point. Thus, the stressing state mode can be expressed by GSED values according to the analytical intentions. Of course, GSED values can be further modeled as other forms of state variables to express structural stressing state characteristic pairs.

For the bottom frame model, the stressing state submodes, the matrixes or vectors formed by GSED values or others, can be composited referring to the locations of strain gauges. For instance, the submodes can be built for individual members, columns, masonry walls, the 1st floor, the 2nd floor, or the frame and wall. All the submodes can combine the stressing state mode of the whole structure. The characteristic parameter can be the sum of the GSED-based elements in the mode. 

### 3.3. Detection of Structural Stressing State Mutation

Structural stressing state characteristic pairs will present the mutation feature at a load level according to the natural law of quantitative change and qualitative change. Here, the Mann–Kendall (M–K) criterion [24,25] is applied to detect the mutation point in the characteristic parameter-load (*E-F*) curve. The operative steps of the M–K criterion are as follows: For the numerical sequence {*E*(*i*)} (the load step *i* = 1, 2, …, *n*), a statistical quantity *d_k_* at the kth load step can be defined as:

(2) $$d_{k}=\sum_{i}^{k}m_{i}\left(2\leq k\leq n\right),\;m_{i}=\left\{\begin{array}{c} +1\\ 0 \end{array}\begin{array}{c} E^{\prime }(i)>E^{\prime }(i)(1\leq j\leq i)\\ otherwise \end{array}\right.$$

where *m_i_* is the cumulative number of the samples; “+1” is to add one more to the present value if the inequality on the right side is satisfied for the *j*th comparison. The mean value and variance of the statistical quantity *d_k_* were calculated as follows:

(3) $$E\left(d_{k}\right)=k\left(k-1\right)/4(2\leq k\leq n),\;Var\left(d_{k}\right)=k\left(k-1\right)\left(2k+5\right)/72\left(2\leq k\leq n\right)$$

Then, a new statistic quantity *UF_k_* is defined by

(4) $$UF_{k}=\left\{\begin{array}{c} 0\\ d_{k}-E\left(d_{k}\right)/\sqrt{Var\left(d_{k}\right)} \end{array}\begin{array}{c} k=1\\ 2\leq k\leq n \end{array}\right.$$

and the *UF_k_-F* curve can be plotted. For the inverse sequence of {*E(j)*} (the load step *j* = *n*, *n* − 1, …,1), the same steps from Equations (2)–(4) are proceeded to derive the *UB_k_-F* curve. Finally, the intersection of the *UF_k_-F* and *UB_k_-F* curves defines the characteristic point of the *E-F* curve, that is, the mutation point of structural stressing state.

## 4. The Stressing State Analysis of the Bottom Frame Model

### 4.1. The Seismic Stressing State Modeling

For the strain values of a point to individual moments in each time history, they can be modeled as the GSED values by Equation (1). For a time history, the GSED values at individual moments can be calculated as:

(5) $$e_{ij}\left(t\right)=\int_{0}^{t}\varepsilon_{ij}\left(t\right)\;dt$$

in which $e_{ij}\left(t\right)$ and $\varepsilon_{ij}\left(t\right)$ are the GSED value and the strain value at the *i*th measured point, at moment *t* and under the *j*th seismic intensity (ground acceleration magnitude) $a_{j}$ , respectively. Since each seismic acceleration during loading includes El Centro, Taft and Wolong waves, and El Centro and Wolong dominate the structural response. e+ is defined as a measure of the stressing state of the measurement points under each seismic conditions. In order to make a difference calculation, the seismic response of the modeling in this study is chosen to be 30 s.

(6) $$e_{ij}^{+}(t)=\frac{\left|e_{i,j}^{\mathrm{El}}(t)-e_{i,j-1}^{\mathrm{Wl}}(t)\right|}{\sum_{i=2}^{7}\left|e_{i,j}^{\mathrm{El}}(t)-e_{i,j-1}^{\mathrm{Wl}}(t)\right|}$$

Correspondingly, the GSED values at $a_{j}$ can be calculated as:

(7) $$e_{ij}^{+}=\int_{0}^{T}e_{ij}^{+}\left(t\right)\;dt$$

in which $e_{ij}^{+}$ is the GSED value at the *i*th measured point during the seismic history T under $a_{j},\;\;a_{j}$ means acceleration at the *j*th earthquake intensity.

**Figure 4:**
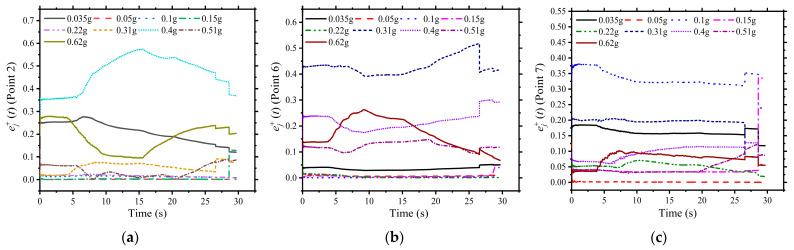
The curves of state variables $e_{2j}^{+}\left(t\right)$ , $e_{6j}^{+}\left(t\right)$ , and $e_{7j}^{+}\left(t\right)$ : (**a**) Point P2, (**b**) Point P6, (**c**) Point P7.

In this test of the bottom frame structure model, only measured points 2~7 recorded the strains in the entire seismic process, and the other 26 points just obtained a part of the strains. For the conventional structural analysis, the strains at the ultimate points could not reflect the working features of the whole structure. However, structural stressing state analysis could effectively reflect the working features of the whole structure using the strains at some specific points, shown in Figure 4 below. 

Besides, the results obtained by selecting different measuring points for structural stressing state analysis are almost identical. This result is because structural stressing state analysis is based on the structural failure law. Here, suppose that the strains at measured points 2~7 could represent the stressing state of the structure, and the stressing state mode ( $M_{j}^{+}$ ) to $a_{j}$ can be built as the vector:

(8) $$M_{j}^{+}={\left[e_{2j}^{+}e_{3j}^{+}\ldots e_{7j}^{+}\right]}^{T}$$

in which superscript “+” represents the strains in the positive seismic direction; if superscript is “−”, it represents the strains in the negative seismic direction. Correspondingly, the stressing state characteristic parameter ( $E_{j}^{+}$ ) can be the sum of several GSED values:

(9) $$E_{j}^{+}=\frac{1}{E_{T}}\sum_{k=2}^{7}e_{kj}^{+}$$

where $E_{T}$ is the maximum among $e_{kj}^{+}$ .

Also, the stressing state submodes for the 1st and 2nd floors can be built as vectors $m_{j}^{I+}$ and $m_{j}^{\mathrm{II}+}$

(10) $$m_{j}^{I+}={\left[e_{2j}^{+}e_{7j}^{+}\right]}^{T}\;\mathrm{or}\;m_{j}^{\mathrm{II}+}={\left[e_{3j}^{+}e_{4j}^{+}e_{5j}^{+}e_{6j}^{+}\right]}^{T}$$

the corresponding characteristic parameters can be written as:

(11) $$E_{j}^{I+}=\frac{1}{E_{T}}\left(e_{2j}^{+}+e_{7j}^{+}\right),\;E_{j}^{\mathrm{II}+}=\frac{1}{E_{T}}\sum_{k=3}^{6}e_{kj}^{+}$$

This study mainly concerns the evolution of the stressing state characteristic pair to the seismic intensities to find out the failure starting point of the bottom frame structure.

### 4.2. The Stressing State Mutation Feature 

The structural stressing state theory characterizes the evolution and mutation in the structural stressing state theory by establishing stressing state mode and characteristic parameters. As a result, there is a great deal of flexibility in how stressing state characteristic pairs are established.

For example, Figure 4 shows the curves for several state variables at individual seismic intensities. It can be seen that the most significant state variables correspond to different seismic intensities, which indicates that the state variables at one measurement point are not representative of the functional characteristics of the whole structure. This is because a single state variable can only model the local state information of a particular measurement point and cannot reflect the global information of the working state of the whole structure. In other words, the study of a single state variable alone cannot reveal the failure characteristics of a structure under seismic conditions.

### 4.3. The Stressing State Features of the Whole Structure

**Figure 5:**
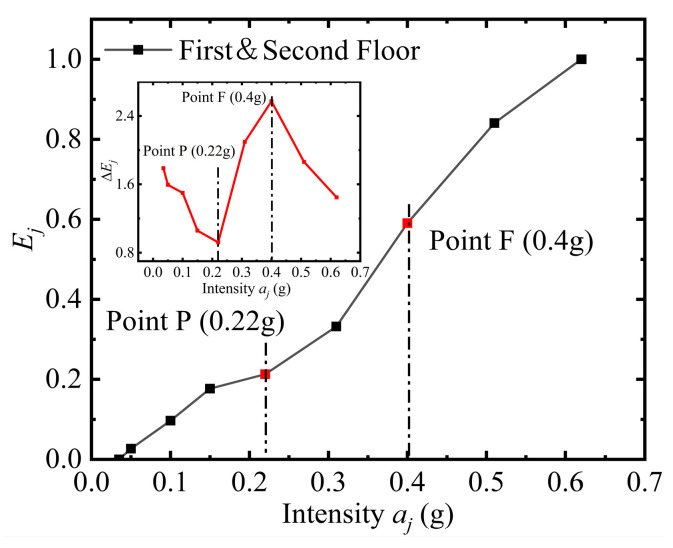
The stressing state mutation features of the bottom frame structure.

Figure 5 shows the *E_j_-a_j_* curves that characterize the overall response of the substructure model. The characteristic points P and Q are defined as the elastoplastic branch (EPB) and the substructure’s failure starting (FS) points under shaking table conditions, respectively. As shown in Figure 5, the seismic intensity/moment of the EPB and FS points are 0.4 g/14.8 s and 0.3 g/7.4 s, respectively, where the mutation around the EPB and FS points are more evident in the Δ*E_j_-a_j_* curves. The Δ*E_j_-a_j_* curve has a Z-shape, with a monotonically decreasing Δ*E_j_-a_j_* curve before the EPB point and a monotonically increasing Δ*E_j_-a_j_* curve after the EPB point until the FS point when it starts to show a decreasing trend.

**Figure 6:**
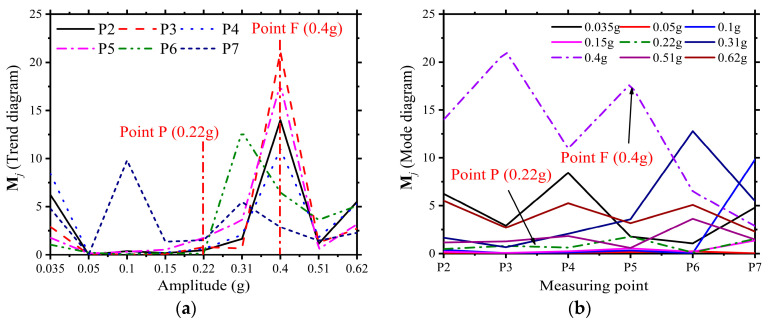
The mutation features of the stressing state mode $M_{j}$ . (**a**) $M_{j}$ (Trend diagram); (**b**) $M_{j}$ (Mode diagram).

The stressing state mode **M***_j_-a_j_* curve corresponding to the *E_j_-a_j_* curve also shows distinctive sharp points and mutations near the failure characteristic points, as shown in Figure 6a,b. The horizontal coordinates of Figure 6a represent the seismic intensity, and the curves represent the different measurement points. The curves show turning and cusp features near the characteristic points P and Q. The horizontal coordinates of Figure 6b represent different measurement points in space, and the curves represent different seismic intensities. The curves show the mode’s leap near the characteristic points. 

These phenomena show that modeling the structure’s stressing state can lead to numerical modes that show significant mutation around the characteristic points, further validating the modeling method and the reasonableness of the characteristic points.

### 4.4. The Stressing State Features of Two Floors

**Figure 7:**
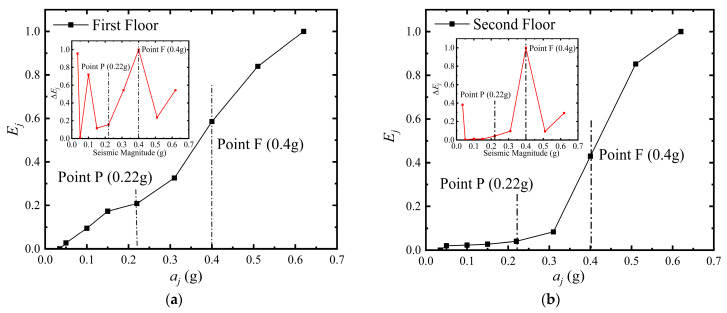
The characteristic curves and the mutation points of the 1st and 2nd floors. (**a**) the 1st floor; (**b**) the 2nd floor.

Figure 7a,b show the characteristic parameter curves, that is, the *E_j_-a_j_* curves, that characterize the stressing state of the two floors of the bottom frame structure model. Combined with the result of M–K criterion and the Δ*E_j_-a_j_* curve, it can be determined that the EPB and FS points of the 1st and 2nd floors of the bottom frame structure are almost identical to the whole stressing state characteristic points, corresponding to a seismic intensity/moment of 0.4 g/14.8 s and 0.3 g/7.4 s. This phenomenon indicates that although the flexibility of the bottom floor characterizes the bottom frame structure, the masonry part of the structure still has good overall working performance during the input of seismic action.

In particular, the Δ*E_j_-a_j_* curve shows a more pronounced turning and cusp characteristic near the EPB and FS points of the bottom frame structure. Moreover, the highest and sharpest points of the Δ*E_j_-a_j_* curve are often found near the FS point, which further demonstrates the importance of the FS point in the seismic design process of the bottom frame structure.

## 5. Discussion

The above stressing state modeling study of the bottom frame structure reveals the EPB and FS points in the failure process by establishing the stressing state mode with characteristic parameters, and further verification can be observed for the experimental phenomena listed in Table 5.

At 0.22 g, the new cracks appeared close to the location of the window bottom, and the last cracks propagated and became cracked all the way through, implying that some limited local failure led to the structural elastic working behavior that started to affect the structural normal working state. Therefore, this point was characterized as the EPB point, and 0.22 g was called the EPB load.

From 0.22 g to 0.40 g, the cracks under the window bottom propagated and formed the small triangle failure area; the cross cracks appeared at the up part of the side beam. The oblique cracks appeared at the masonry wall close to the pedestal of the bottom frame. The local failure quickly propagated to present the structural elastoplastic working state, that is, the structure worked in the plastic formation accumulation state, which the structural design requirement could not allow.

At 0.40 g, the new cracks appeared under the window bottom, and the previous ones propagated, further promoting the triangle failure area. The oblique cracks developed at the masonry wall close to the pedestal of the bottom frame, together with new cracks. The structural stressing state form would mutate to the other, lose the normal working state, and start its failure. A load of 0.40 g was defined as the structural failure load in structural stressing state theory. It should be stated again that 0.40 g was the structural failure starting point and the embodiment of the structural failure law. Furthermore, the EPB point could be the principle derived from structural failure law, which might be taken as the general design principle of structures.

So far, we can summarize that the bottom frame structure indeed presents the stressing state mutation behavior at a certain seismic intensity, complying with structural failure law or the natural law of quantitative change to qualitative change. In other words, when the structural stressing state quantitatively develops to a certain extent, it will qualitatively mutate and present a different profile (stressing state mode) from the previous one, which the M–K criterion can detect. Then, based on the structural failure starting point, it can detect the structural EPB characteristic point, which might be taken as the design principle. The EPB point provides the physical-law-based reference to improving the design of bottom frame structures or other structures. 

So far, we can summarize that the substructure does exhibit a mutation in stressing state behavior under specific seismic intensities, in line with the structural damage law or the quantitative to qualitative change law. In other words, when the quantitative change in the structural stressing state develops to a certain level, a qualitative change will occur, presenting a different shape from the previous one. Combining the results of the M–K criterion and the mutation characteristics of the Δ*E_j_-a_j_* curve, the FS point, and EPB point can be detected. Among other things, the EPB point provides a reference for improving the design of bottom frame structures or other structures based on physical laws.

For comparison’s sake, the structural stressing state feature provides a new foothold complying with the natural law for structural analysis and design, different from the foothold of the structural ultimate working state, which is the existing structural analysis and design standard. The two footholds have the essential difference, one in particular and general; the other is uncertain and specific. In a sense, structural analysis and design have been anticipating and pursuing the former, but the foothold on the structural ultimate state would lead to the belief that there was a physical law for structural bearing capacity. This belief may have been broken by discovering the starting point of the structure’s failure process: the specific embodiment of the natural law in the structural working process. Thus, structural analysis and design could be mainly governed by the definite and general structural working law (structural failure law), that is, the structural failure starting point and the EPB point with the attribute of certainty, rather than the structural failure ending point (structural ultimate state) with uncertainty/randomness. 

## 6. Conclusions

At present, the application of structural stressing state theory to various structures has significance in science and engineering: the scientific significance is to achieve the specific scientific discovery in the working process of a structure or a type of structure based on the natural law from quantitative change to qualitative change of a system, that is, to reveal the general and definite working law of the structures unseen in the existing structural analysis; the engineering significance is it addresses the classic issue, the uncertainty of structural bearing capacity and the inconsistent design criterion of various structures. In this study, structural stressing state theory is first applied to reveal the seismic working law of the bottom frame structure, from which can be drawn the following conclusions: 

The GSED values transferred from the experimental strain data can express the substructure’s stressing state mode and characteristic parameters under seismic action. The M–K criterion and the Δ*E_j_-a_j_* curve can find two mutation points of stressing states, the starting point of the structural damage process and the elastoplastic branching point during the regular operation of the structure. The seismic capacity of the bottom frame structure should be determined as the failure starting point of the structural damage process, and the seismic intensity can be referred to as the structural damage load. The EPB point can be used as a direct reference for the design of the substructure as a design criterion extracted from the laws of nature or the structural damage law. The method based on the structural stressing state theory can eliminate the typical problem of inherent randomness in the ultimate state, which can lead to explicit design criteria for the seismic load capacity of the structure.

In addition, this study has developed a method for modeling experimental seismic strain data and analyzing the characteristics of structural seismic stress states, enriching and developing structural stress state theory and facilitating its further application.

## Figures and Tables

**Table 1:** The information of the tested bottom frame structure (mm).

Masonry wall height: 3300, thickness: 240	MU10 clay brick
Frame height: 4500	Mortar: M5
Section of column: 400 × 400	Concrete: C30
Section of longitudinal beam: 300 × 400	Aseismic protection; 7 class
Section of lateral beam (side span): 300 × 600	Ground acceleration 0.1 g
Section of lateral beam (middle span): 300 × 400	Class II venues
Section of constructional column: 240 × 240	Serve time: 50 years

**Table 2:** The similarity of physical parameters.

Parameter	Length	Elastic Modulus	Acceleration	Period	Velocity	Equivalent Density	Mass
Symbol	*l_r_*	*E_r_*	*a_r_*	*T_r_* = (*l_r_*/*a_r_*)^1/2^	*v_r_* = (*l_r_* × *a_r_*)^1/2^	*ρ_r_* = *E_r_*/(*l_r_* × *a_r_*)	*E_r_ × l_r_* ^2^ */a_r_*
Ratio	1/5	1/3	1	1/2.23	1/2.23	1.67	1/75

**Table 3:** The mass parameters of individual stories and the artificial weights.

Item	Real Volume (m^3^)	Density (m^3^)	Real Mass (t)	Mass of Model (t)
Concrete on the 1st floor	61.881	2.5	154.702	1.238
Concrete on the 2nd floor	19.703	2.5	49.257	0.394
Masonry on the 2nd floor	84.326	1.8	151.787	1.214
Concrete on the 3rd floor	19.703	2.5	49.257	0.394
Masonry on the 3rd floor	84.326	1.8	151.757	1.214
Concrete on the 4th floor	23.094	2.5	57.735	0.462
Masonry on the 4st floor	83.799	1.8	150.839	1.208
Live load			137.665	
Total	376.833		903.031	6.123
Artificial weights	*m*_T_ = 6.123 t, *m* = 12.040 t; *m*_w_ = 5.917 t.			

**Table 4:** The parameters of the shaking table and the seismic inputs.

The Parameters of the Shaking Table			
Load Capacity (t)	Size (m)	Horizontal & Vertical Displacements (cm)	Horizontal & Vertical Acceleration (m/s^2^)
30	5 × 5	8, 10	5, 7

**Table 5:** The seismic input scheme and the failure profiles of the bottom frame structure.

Case	Description and Profile of Typical Failure (along X-Axis)
T1 (0.035 g–7 dg) T2 (0.055 g–7.5 dg)	Under the El, Tf, and Wl waves with seismic intensities of 0.035~0.10 g, no visible cracks occurred.
T3 (0.10 g–7 dg)	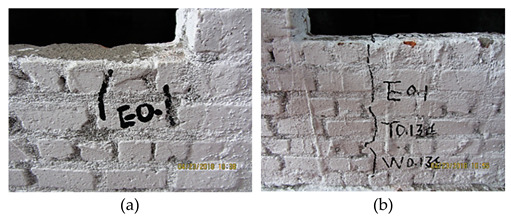 The masonry close to the window bottom first cracked under the El wave (a) and then slightly propagated under Tf and Wl waves (b).
WNS (0.07 g) T4 (0.15 g–7.5 dg) El, Tf, Wl	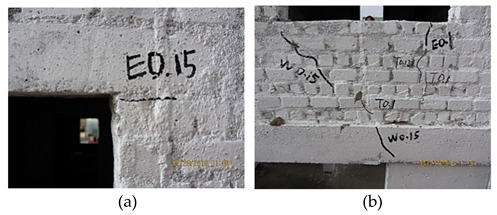 Slight cracks appeared at the location of the window contact with the lintel (a). The oblique crack appeared from the window corner to the side beam (b).
WNS (0.10 g) T5 (0.22 g–7 dg) El, Tf, Wl	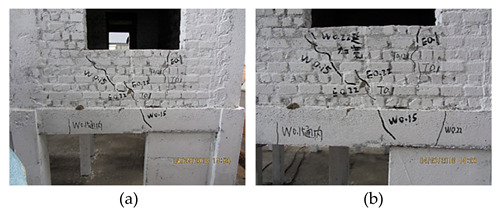 The new cracks appeared close to the location of the window bottom (a). The previous cracks propagated and cracked all the way through (b).
WNS (0.10 g) T6 (0.31 g–7 dg) El, Tf, Wl	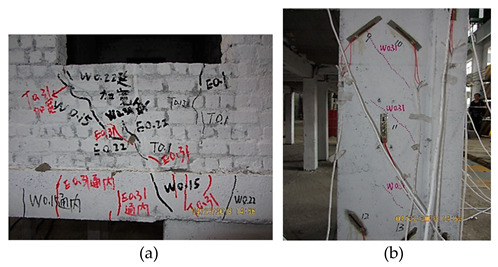 The cracks under the window bottom propagated and formed the slight triangle failure area; the cross cracks appeared at the upper part of the side beam (a). The oblique cracks appeared at the masonry wall close to the pedestal of the bottom frame (b).
WNS (0.10 g) T7 (0.40 g–8 dg) El, Tf, Wl	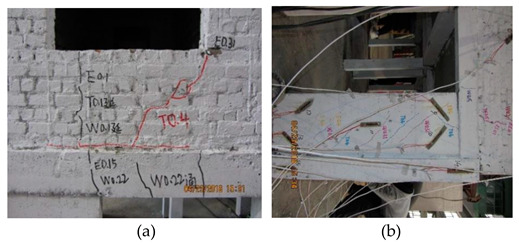 The new cracks appeared under the window bottom, and the previous cracks propagated, which further promoted the triangle failure area (a). The oblique cracks developed at the masonry wall close to the pedestal of the bottom frame together with new cracks (b).
WNS (0.10 g) T8 (0.51 g–8 dg) El, Tf	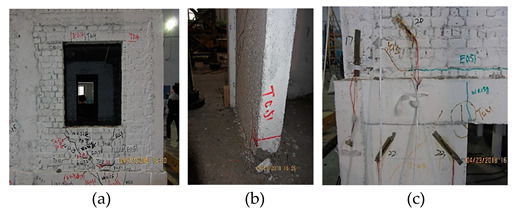 All the previous cracks propagated (a). The oblique cracks under the window bottom cracked all the way through with those on the beam and formed the plastic hinge. The concrete dropped at the bottom corners of the masonry wall (b). The horizontal cracks appeared and propagated along the contacting section between the masonry on the 2nd floor and the bottom frame (c).
WNS (0.10 g) T9 (0.62 g–9 dg) El, Tf WNS (0.10 g)	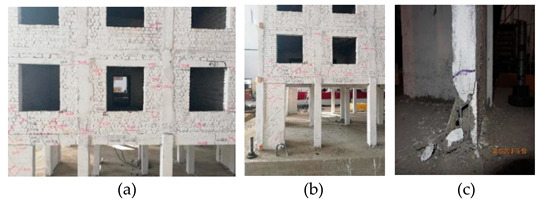 All the previous cracks further propagated failed; the cracks appeared at the window edge on the 3rd floor (a). The cracks came through along the lintel to the structural column (b). The steel bar in the bottom column yielded together with the concrete drop (c).

**Table 6:** Inter-story displacement angle (EL-Centro).

Inter-Story Displacement Angle (EL-Centro)		1st Floor		2nd Floor		3rd Floor		4th Floor	
		X	Y	X	Y	X	Y	X	Y
Acceleration peak (g)	0.035	0.0012	0.0008	0.0018	0.0013	0.0012	0.0015	0.0010	0.0016
	0.1	0.0007	0.0007	0.0009	0.0021	0.0014	0.0018	0.0011	0.0012
	0.22	0.0029	0.0017	0.0034	0.0033	0.0027	0.0036	0.0031	0.0043
	0.31	0.0033	0.0018	0.0025	0.0042	0.0032	0.0039	0.0033	0.0037
	0.4	0.0064	0.0018	0.0033	0.0039	0.0018	0.0047	0.0022	0.0038
	0.51	0.0136	0.0032	0.0049	0.0031	0.0025	0.0041	0.0028	0.0051
	0.62	0.0201	0.0077	0.0050	0.0048	0.0029	0.0049	0.0031	0.0028

**Table 7:** Inter-story displacement angle (Taft).

Inter-Story Displacement Angle (Taft)		1st Floor		2nd Floor		3rd Floor		4th Floor	
		X	Y	X	Y	X	Y	X	Y
Acceleration peak (g)	0.035	0.0010	0.0007	0.0014	0.0013	0.0009	0.0020	0.0009	0.0020
	0.1	0.0008	0.0009	0.0012	0.0014	0.0012	0.0017	0.0012	0.0014
	0.22	0.0028	0.0013	0.0025	0.0016	0.0015	0.0022	0.0020	0.0034
	0.31	0.0047	0.0022	0.0026	0.0029	0.0020	0.0022	0.0020	0.0021
	0.4	0.0085	0.0025	0.0030	0.0039	0.0023	0.0046	0.0022	0.0039
	0.51	0.0085	0.0044	0.0030	0.0046	0.0023	0.0067	0.0022	0.0032
	0.62	0.0207	0.0132	0.0043	0.0037	0.0022	0.0060	0.0023	0.0030

**Table 8:** Inter-story displacement angle (Wolong).

Inter-Story Displacement Angle (Wolong)		1st Floor		2nd Floor		3rd Floor		4th Floor	
		X	Y	X	Y	X	Y	X	Y
Acceleration peak (g)	0.035	0.0008	0.0007	0.0009	0.0009	0.0009	0.0019	0.0010	0.0017
	0.1	0.0008	0.0007	0.0011	0.0011	0.0012	0.0013	0.0010	0.0013
	0.22	0.0024	0.0012	0.0025	0.0024	0.0013	0.0032	0.0017	0.0021
	0.31	0.0034	0.0014	0.0022	0.0016	0.0021	0.0022	0.0015	0.0025
	0.4	0.0081	0.0022	0.0024	0.0014	0.0015	0.0021	0.0017	0.0025

## Data Availability

The data presented in this study are available on request from the corresponding author.
