# Correction: Zhang et al. The Stressing State Features of a Bottom Frame Structure Revealed from the Shaking Table Strain Data. *Materials* 2023, *16*, 1809

**DOI:** 10.3390/ma17010169

**Published:** 2023-12-28

**Authors:** Lingxin Zhang, Rui Li, Zijie Shen, Bai Liu, Jianhui Kong, Guangchun Zhou

**Affiliations:** 1 Key Laboratory of Earthquake Engineering and Engineering Vibration, Institute of Engineering Mechanics, China Earthquake Administration, Harbin 150086, China; lingxin_zh@126.com (L.Z.); jianh_kong@163.com (J.K.); 2 Key Laboratory of Earthquake Disaster Mitigation, Ministry of Emergency Management, Harbin 150086, China; 3 School of Civil Engineering, Harbin Institute of Technology, Harbin 150090, China; liubai_12138@163.com (B.L.); gzhou@hit.edu.cn (G.Z.); 4 Key Lab of Structures Dynamic Behavior and Control of China Ministry of Education, Harbin 150090, China; 5 Key Lab of Smart Prevention and Mitigation of Civil Engineering Disasters of the Ministry of Industry and Information Technology, School of Civil Engineering, Harbin Institute of Technology, Harbin 150090, China

In the original publication [1], there was a mistake in Figure 1 and Figure 2, and Table 3 as published. Because of the authors’ negligence, the photo and schematic of the bottom frame structure shown in the original publication’s Section 2 were confused with another similar experiment by our research group. The mistakes in Table 3 are the same. The corrected Figure 1 and Figure 2, and Table 3 are shown below. The authors state that the scientific conclusions are unaffected. This correction was approved by the Academic Editor. The original publication has also been updated.

## Figures and Tables

**Figure 1:**
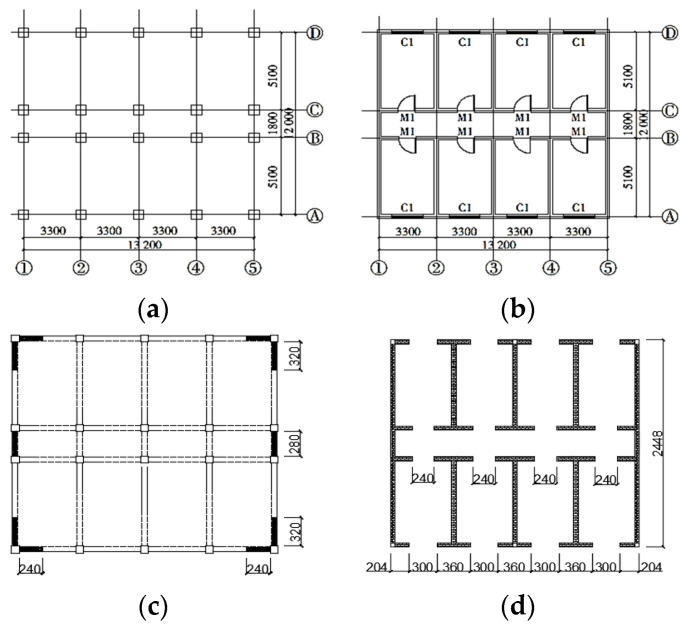
The floor plans of the bottom frame structure: (**a**) the bottom floor plan, (**b**) the standard story plan, (**c**) the anti-seismic wall plan in the bottom story, (**d**) the constructional columns in the upon stories.

**Figure 2:**
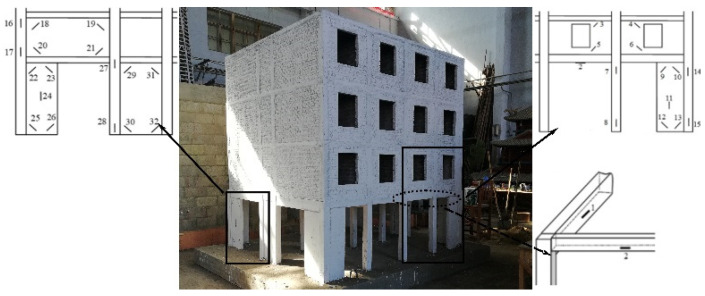
The bottom frame model, the layout of strain gauges and the cracking.

**Table 3:** The mass parameters of individual stories and the artificial weights.

Item	Real Volume (m^3^)	Density (m^3^)	Real Mass (t)	Mass of Model (t)
Concrete on the 1st floor	61.881	2.5	154.702	1.238
Concrete on the 2nd floor	19.703	2.5	49.257	0.394
Masonry on the 2nd floor	84.326	1.8	151.787	1.214
Concrete on the 3rd floor	19.703	2.5	49.257	0.394
Masonry on the 3rd floor	84.326	1.8	151.757	1.214
Concrete on the 4th floor	23.094	2.5	57.735	0.462
Masonry on the 4st floor	83.799	1.8	150.839	1.208
Live load			137.665	
Total	376.833		903.031	6.123
Artificial weights	*m*_T_ = 6.123 t, *m* = 12.040 t; *m*_w_ = 5.917 t.			
